# Walking-Speed-Adaptive Gait Phase Estimation for Wearable Robots

**DOI:** 10.3390/s23198276

**Published:** 2023-10-06

**Authors:** Sanguk Choi, Chanyoung Ko, Kyoungchul Kong

**Affiliations:** Department of Mechanical Engineering, Korea Advanced Institute of Science and Technology (KAIST), Daejeon 34141, Republic of Korea; sangukchoi@kaist.ac.kr (S.C.); kkyle0927@kaist.ac.kr (C.K.)

**Keywords:** gait phase estimation, inertial measurement unit, wearable robots

## Abstract

This paper introduces a Gait Phase Estimation Module (GPEM) and its real-time algorithm designed to estimate gait phases continuously and monotonically across a range of walking speeds and accelerations/decelerations. To address the challenges of real-world applications, we propose a speed-adaptive online gait phase estimation algorithm, which enables precise estimation of gait phases during both constant speed locomotion and dynamic speed changes. Experimental verification demonstrates that the proposed method offers smooth, continuous, and repetitive gait phase estimation when compared to conventional approaches such as the phase portrait method and time-based estimation. The proposed method achieved a 48% reduction in gait phase deviation compared to time-based estimation and a 48.29% reduction compared to the phase portrait method. The proposed algorithm is integrated within the GPEM, allowing for its versatile application in controlling gait assistive robots without incurring additional computational burden. The results of this study contribute to the development of robust and efficient gait phase estimation techniques for various robotic applications.

## 1. Introduction

Walking, which imparts mobility through repetitive lower-limb movements, is one of the essential activities in human life. To enhance or assist the mobility of human, many researchers have expressed great interest in development of gait assistive robots, such as exoskeletons [1,2,3,4,5], soft exosuits [5,6,7,8,9,10,11], and robotic prostheses [12,13,14].

Recent advances in these robots have led to the development of real-time gait phase estimation methods. Portable sensor systems and online algorithms have been employed for this purpose. One of the most effective methods involves using foot pressure data obtained through force sensing resistors or pneumatic sensors [15,16,17]. The approach enables the classification of stance and swing phases and the estimation of subdivided gait events. However, these sensors lack durability due to repetitive impacts from the ground and cannot provide meaningful information during the swing phase, which constitutes approximately half of the gait cycle.

In recent years, there has been a growing trend in research to predict the gait phase using continuous and intuitive kinematic data [18,19,20,21,22,23,24,25]. Kinematic data can be obtained through encoders attached to exoskeletal joints [18,19,20], IMUs located on human lower-limb segments [4,21,22,23], or by fusing both [24,25]. An adaptive Frequency Oscillator (AFO) has been implemented to estimate the current phase of repetitive human motion, i.e., the gait phase [8,18,26,27,28]. Especially, Some researchers have improved the performance of the AFO-based algorithm by adopting a function that resembles the actual joint angle as a basis function instead of a simple sinusoidal function [26]. However, these methods require individual tuning and are challenging to apply to real robot applications because if the joint angle pattern changes, it can be divergent in the worst case. Machine learning (ML) technology has been actively incorporated into gait phase estimation studies [24,25,29,30]. However, ML-based gait phase estimation requires a large amount of data to ensure accuracy, and accuracy may be limited when the subjects change.

The use of phase portrait is an alternative approach for estimating the gait phase. This method plots a joint angle and its differential value or integral value each on the x-axis and y-axis to create a circular-shaped graph(portrait) that represents a user’s walking pattern. Consequently, the gait phase can be estimated as the phase of the portrait [12,21,31,32,33]. The many studies have used low pass filtered joint angle, not the raw data since the raw data contain the unexpected disturbance due to the ground contact or the misalignment between the sensor and the body segment [12,21,31,32]. The filters cause an inevitable phase lag which varies depending on the gait frequency. Consequently, the gait phase can be distorted, and its consistency can be reduced over varying gait speed conditions. It can be a critical problem in real-world applications.

This paper proposes a speed-adaptive online gait phase estimation algorithm based on the phase portrait method using the thigh flexion/extension angle. To estimate an undistorted gait phase in real-time, we introduce a gait frequency adaptive low-pass filter (GFAF), which maintains a consistent phase delay regardless of the gait speed. This consistent phase delay introduced by GFAF can be easily compensated for. Another consideration of the phase portrait-based method is the smoothness of the gait phase. The normalization process is needed to unify the two variables on the x and y axes. In the real-time algorithm, data such as the maximum/minimum values of the kinematic data in the previous stride are utilized and updated in every stride during the normalization process. When the update occurs, the phase portrait and the gait phase can become discontinuous at varying walking speeds. To ensure a smooth gait phase, we revise the normalization process in this paper. Moreover, to implement the online gait phase algorithm, we design a hardware module for gait phase estimation.

## 2. Offline Gait Phase Portrait Method

**Figure 1:**
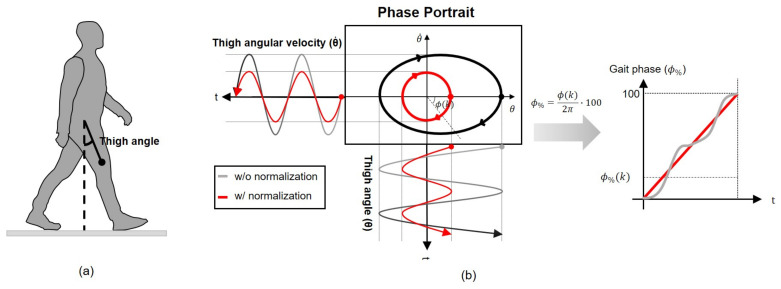
Basic principle of the gait phase estimation algorithm. (**a**) Periodic nature of thigh movement in gait. The thigh angle in the sagittal plane is utilized to construct a phase portrait. (**b**) Gait phase estimation method based on the phase portrait.

The gait phase estimation algorithm employed in this paper is based on the phase portrait of the thigh angle. A phase portrait is a visual representation of a system’s trajectory in the phase plane, where the two axes depict the values of its two state variables. In this paper, the system is the human gait system, and we employ the thigh angle and its differentiation as the two state variables. The thigh angle (θ) is defined in the sagittal plane with respect to the ground’s normal line, with positive values representing flexion, as illustrated in Figure 1a. The phase portrait of a gait is constructed by setting the thigh angle as the x-axis and the angular velocity of the thigh as the y-axis.

Although detailed movements may vary depending on subjects, gait environments, and strides, from a macroscopic perspective, the thigh exhibits periodic movement during gait. This periodicity is applicable to individuals with different gait types, including normal and abnormal gaits. In all gait patterns, the thigh movement generates periodic outputs, including angle and angular velocity. Figure 1b illustrates the gait phase estimation algorithm based on the phase portrait. When the thigh angle and thigh angular velocity in the time domain are transformed into the phase plane, a phase portrait represented by the black solid line moves periodically in a clockwise direction. Using this plot, the gait phase can be estimated as the angle with respect to the x-axis of the portrait, which is calculated as

(1) $$\phi \left(k\right)=\left\{\begin{array}{cc} -\arctan(\dot{\theta }\left(k\right)/\theta \left(k\right)), & \mathrm{if}\;\theta \left(k\right)\geq 0,\dot{\theta }\left(k\right)\leq 0\\ -\arctan(\dot{\theta }\left(k\right)/\theta \left(k\right))+\pi , & \mathrm{if}\;\theta (k)<0\\ -\arctan(\dot{\theta }\left(k\right)/\theta \left(k\right))+2\pi , & \mathrm{otherwise} \end{array}\right.$$

where ϕ(k)∈[0,2π) represents the gait phase. Negative signs are applied to ϕ(k) in (1) to denote the clockwise direction as positive. For simplicity, the gait phase ϕ in radians is converted to a percentage, i.e., $\phi_{\%}\left(k\right)=50\phi \left(k\right)/\pi$.

A phase portrait ideally should be a circle centered at the origin to ensure that the gait phase remains linear. However, due to differences in axis scales and data biases, the portrait often appears as an ellipse with a biased center, as indicated by the black line in Figure 1b. To prevent distortion in gait phase estimation, a normalization process is crucial for two purposes: shifting the center of the portrait to the origin and unifying the scales of the axes. The normalized angle and angular velocity are computed by

(2) $$\theta_{normalized}\left(k\right)=\frac{\theta \left(k\right)-\theta_{o}\left(k\right)}{A_{\theta }\left(k\right)},$$

and

(3) $${\dot{\theta }}_{normalized}\left(k\right)=\frac{\dot{\theta }\left(k\right)-{\dot{\theta }}_{o}\left(k\right)}{A_{\dot{\theta }}\left(k\right)}$$

where $\theta_{o}\left(k\right)$ and $\dot{\theta }o\left(k\right)$ represent the center values of θ(k) and $\dot{\theta }\left(k\right)$, while $A_{\theta }\left(k\right)$ and $A_{\dot{\theta }}\left(k\right)$ are the amplitudes of θ and $\dot{\theta }$ in the current stride, respectively. The normalized angle and angular velocity transform the phase portrait into a unit circle centered at the origin, as indicated by the red circle in Figure 1b.

While heel strike is commonly used as the initial gait event in the gait cycle, at the 0% point of the cycle, some studies estimating gait phase based on thigh kinematics have adopted the maximum hip flexion or extension as the initial gait event [8,22,25]. In this study, we have set the maximum hip flexion as the starting point at 0% of the gait cycle.

## 3. Conversion to Online Algorithm

### 3.1. Gait Phase Estimation Module

**Figure 2:**
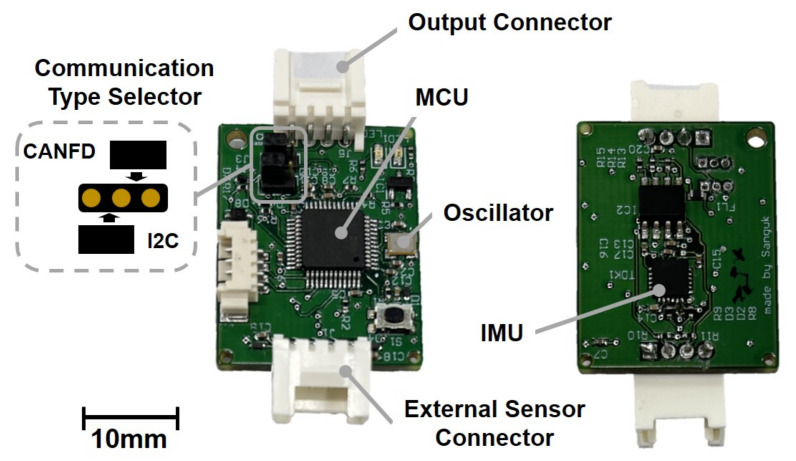
Configuration of a gait phase estimation module(GPEM).

To integrate the online gait phase estimation algorithm, we designed a gait phase estimation module (GPEM), as depicted in Figure 2. The GPEM features a compact and lightweight form factor, weighing only 9.2 g and measuring 29×33×10.5 mm. Its primary components include a MEMS IMU and a microcontroller (MCU). The GPEM acquires raw data from the IMU and processes gait phase estimation via the MCU. The estimated gait phase is then transmitted to the robot controller and utilized to control the human assistive robot. Since GPEM performs gait phase estimation tasks on behalf of the robot controller, it can reduce the computational load on the robot controller.

For the embedded IMU in the GPEM, we have adopted the MPU-6050 chip, which comprises a 3-axis gyroscope and a 3-axis accelerometer. Although the IMU provides kinematic data via its onboard processor, the GPEM utilizes the raw acceleration and angular velocity data from the IMU. It then processes thigh angle estimation, tailoring the algorithm specifically for gait analysis. The algorithm is embedded in the MCU, an STM32G431, which operates with an external crystal oscillator running at a frequency of 12 MHz. Data from the IMU are transmitted to the MCU via the SPI protocol, with a sampling frequency set at 1 kHz.

The GPEM offers versatility through two communication protocols: $I^{2}C$ and CAN FD. Switching between these protocols is easily achieved by configuring a pin on a communication type selector, as demonstrated in Figure 2. Furthermore, the GPEM can interface with external sensors, such as ground reaction force sensors and myoelectric sensors.

### 3.2. Overall Algorithm

**Figure 3:**
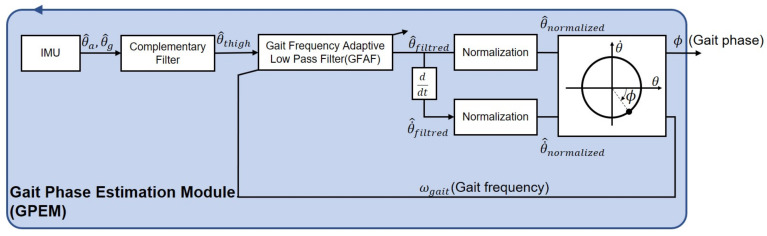
Overall algorithm of the speed-adaptive gait phase estimation implemented in the gait phase estimation module (GPEM).

The proposed online gait phase estimation algorithm embedded on the GPEM is presented in Figure 3. In order to enable real-time implementation, all algorithms operating in the continuous-time domain are converted to the discrete-time domain using backward approximation.

The thigh angle in the sagittal plane is determined through sensor fusion of acceleration and gyroscope data, obtained from the IMU embedded on the GPEM. The complementary filter is used for this purpose [34,35,36]. Only information within a reliable frequency range is extracted from each sensor. To mitigate external disturbances resulting from ground impact at the heel strike, the estimated thigh angle undergoes low-pass filtering. The low-pass filter is designed to avoid the distortion of the gait phase estimation over various walking speeds. Subsequently, normalization of both the angle and angular velocity is performed to ensure that the phase portrait resembles a unit circle. Based on the phase portrait of the normalized data, real-time estimation of the gait phase is achieved.

### 3.3. Gait Frequency Adaptive Filter

Even though the thigh angle was estimated by the complementary filter, the disturbance due to ground impact at the initial contact and a misalignment between the thigh and the GPEM is also included in the estimation. Especially, the angular velocity of the thigh, which is calculated by numerical differentiation of the angle, is heavily affected by the high-frequency noise. For this purpose, applying a low-pass filter is a suitable method to eliminate these noise and disturbance, resulting in a smooth plot of the angle and the angular velocity. A Butterworth low pass filter is applied for this purpose, and the transfer function of the filter is

(4) $$LPF\left(j\omega \right)={\left(\frac{\omega_{c}}{j\omega +\omega_{c}}\right)}^{n}$$

where $\omega_{c}$ is the cutoff frequency of the filter and *n* is the order of the filter. The low pass filter can have a smoothing effect on the raw data, but the phase delay is inevitable. Since the phase delay of (4) is derived as

(5) $$phase\left(\omega \right)=-n\arctan\left(\omega /\omega_{c}\right),$$

and it depends on the frequency of the data.

**Figure 4:**
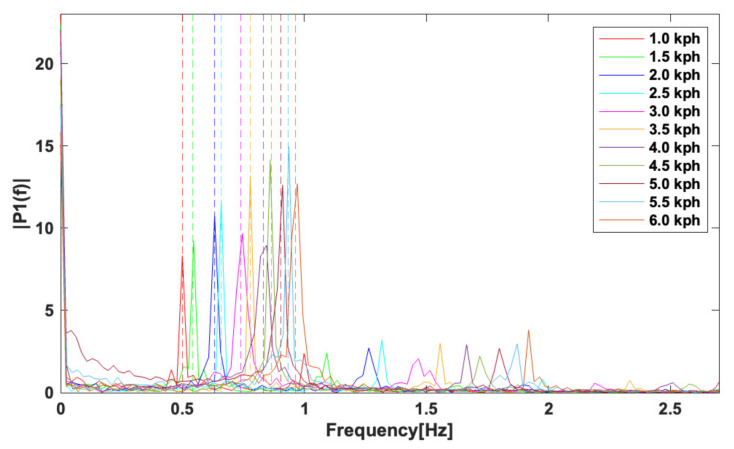
Fast Fourier Transform (FFT) analysis of thigh angle with various walking speeds and estimation of gait frequencies. Solid lines and dashed lines indicate the frequency responses of the thigh and the estimated gait frequencies, respectively.

The dominant frequency of the thigh angle changes regarding to gait speed as shown in Figure 4. It means that applying a low pass filter can cause the inconsistent phase delay over the walking speeds, which leads to the distortion of the gait phase estimation. To improve the accuracy of the gait phase estimation regardless of the gait speed, a gait frequency adaptive low pass filter(GFAF) is proposed. A cutoff frequency of the proposed filter is proportional to gait frequency as

(6) $$\omega_{c}=\alpha \omega_{gait}$$

where α is a proportional factor that can be tuned and $\omega_{gait}$ is the dominant frequency of gait, defined as

(7) $$\omega_{gait}=\frac{2\pi }{T_{stride}}.$$

$T_{stride}$ is the time elapsed between the first contact of two consecutive footsteps of the same foot.

To verify (7) is reasonable for the gait frequency, a walking test was performed. The dominant frequencies of the thigh angle and the angular velocity were analyzed for various walking speeds, from 1.0 km/h to 6.0 km/h in Figure 4. It is shown that the dominant frequency increases with increasing the gait speed. At each gait speed, the thigh angle was converted into a frequency domain using a fast Fourier transform (FFT) tool equipped in MATLAB, resulting in solid lines in Figure 4. The gait frequencies estimated by (7) are represented as dotted vertical lines in the same figure. The root mean square error (RMSE) between the measured and estimated gait frequency was 0.674%, which means that the estimation is reasonable.

The phase of the proposed filter at the gait frequency $\omega_{gait}$ is

(8) phase(ω)=−narctan(1/α).

Note that the phase only depends on the order of the filter, *n*, and the proportional factor, α. Therefore, if *n* and α are the constant, The phase delay at $\omega_{gait}$ is constant regardless of the gait frequency. The filtered gait phase is compensated the phase delay due to the GFAF as

(9) $$\phi_{\mathrm{filtered}}\left(k\right)=\phi \left(k\right)+n\arctan\left(1/\alpha \right).$$

**Figure 5:**
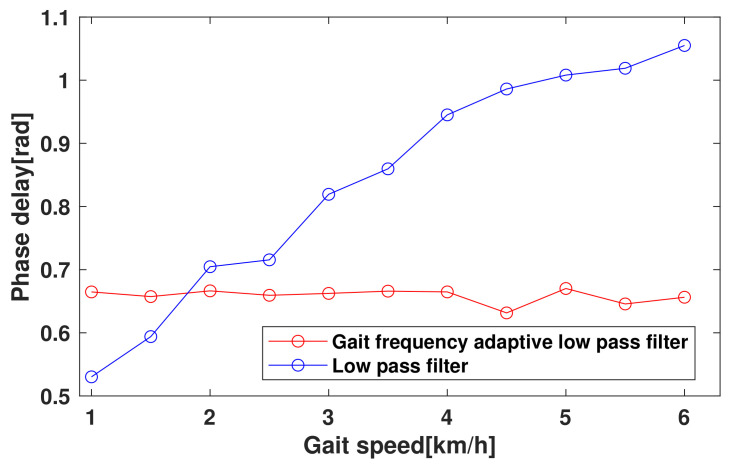
Phase delay comparison between gait frequency adaptive filter (red) and low-pass filter (blue) over various walking speeds.

Figure 5 shows the comparison of the phase delay of the thigh angle due to the GFAF with a proportional factors, α=2.5 and a low pass filter with a cutoff frequency of 1.5 Hz at speeds ranging from 1.0 km/h to 6.0 km/h. The GFAF with α=2.5 showed a nearly constant phase delay regardless of walking speed, while the LPF showed a significant increase in phase delay as the walking speed increased. The maximum differences in phase delay of the LPF and GFAF were 0.54 rad and 0.02 rad, respectively, indicating that the LPF caused a maximum gait phase distortion of 8.59%, while the GFAF only caused 0.32%. There exists a small error between the phase delays by the theoretical calculation and the experimental measurement; The mean of phase delay of the GFAF is 0.659 rad, whereas the theoretical phase delay by (8) is 0.761 rad. The result suggests that the GFAF provides a more accurate and consistent measurement of thigh angle than the LPF, particularly at varying walking speeds, and offers a promising alternative.

### 3.4. Normalization Parameter Estimation

To convert the gait phase estimation algorithm into real-time application, the normalization process should be revised. As mentioned in Section 2, the normalization parameters such as the center and amplitude are used in the normalization. However, the normalization parameters are not constant in real-world due to the changes of walking speed or stride length.

**Figure 6:**
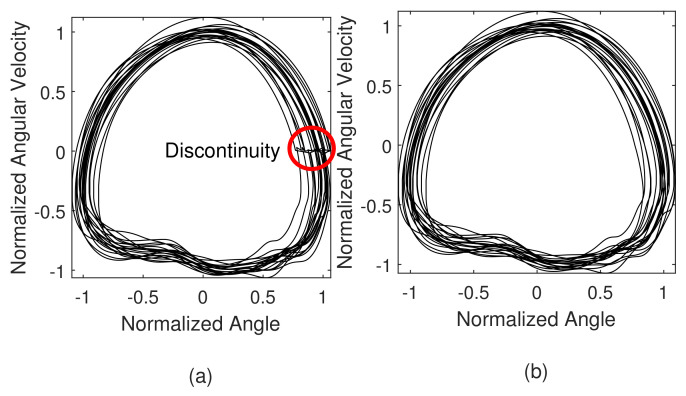
Real-time normalization results. (**a**) discretely updating the normalization parameters causes discontinuity in the phase portrait. (**b**) By using the filtered normalization parameters, the discontinuity is disappeared.

The centers and amplitudes of the thigh angle and the thigh velocity are calculated by

(10) $$\theta_{o}\left(k\right)=\frac{\theta_{max}\left(k\right)+\theta_{min}\left(k\right)}{2},$$

(11) $${\dot{\theta }}_{o}\left(k\right)=\frac{{\dot{\theta }}_{max}\left(k\right)+{\dot{\theta }}_{min}\left(k\right)}{2},$$

(12) $$A_{\theta }\left(k\right)=\frac{\theta_{max}\left(k\right)-\theta_{min}\left(k\right)}{2},$$

and

(13) $$A_{\dot{\theta }}\left(k\right)=\frac{{\dot{\theta }}_{max}\left(k\right)-{\dot{\theta }}_{min}\left(k\right)}{2},$$

individually, where $\theta_{max}$, $\theta_{min}$, ${\dot{\theta }}_{max}$ and ${\dot{\theta }}_{min}$ are maximum or minimum values of θ or $\dot{\theta }$ in the previous stride. The maximum and minimum data are updated when the thigh angle is the maximum(maximum flexion) because there is no impact from the ground so that the data are not distorted and easy to distinguish. However, updating the parameter discretely at the update point results in a discontinuous phase portrait, as shown in Figure 6a. Consequently, the gait phase is estimated with reversed and discrete points. This causes a fatal problem with gait phase-based control. To prevent this problem, a first order infinite impulse filter was applied to the normalization parameter;

(14) $$H\left(z\right)=\frac{1-a}{1-az^{-1}}$$

where *a* is a pole of the filter such that a∈[0,1). The pole was set as 0.98 so that the phase portrait has a smooth and continuous manner as illustrated in Figure 6b.

## 4. Experimental Validation

### 4.1. Experimental Protocols

**Figure 7:**
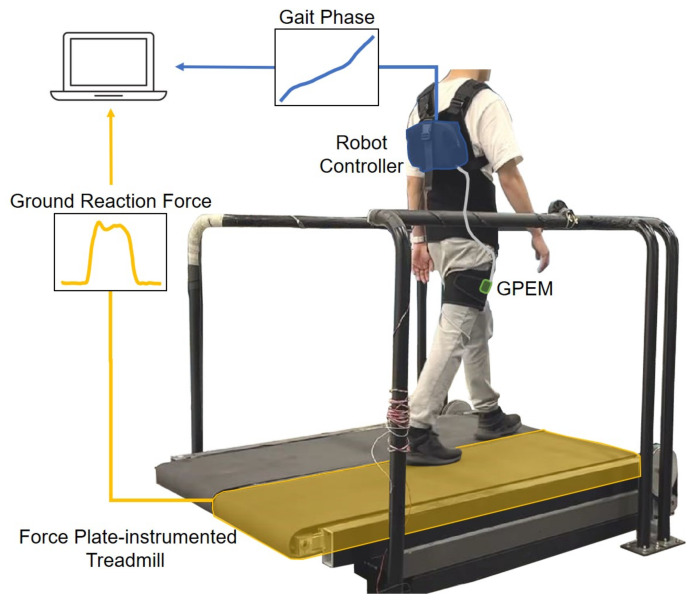
Experimental setup for data collection. The data, the gait phase from the GPEM and the ground reaction force from the treadmill, were collected. To synchronize the data, subjects were instructed to impact the ground at the beginning of each trial.

Walking experiments were conducted to validate the proposed online gait phase algorithm. The experiment received approval from the Institutional Review Board (IRB) at the Korea Advanced Institute of Science and Technology (KAIST). Eleven healthy subjects were recruited for the study, comprising eight males and three females. These participants exhibited an average age of 25.73±1.35 years, an average height of 171.09±7.75 m, and an average body weight of 65.1±11.1 kg. They were instructed to walk on a force plate-instrumented treadmill while wearing a lightweight soft wearable robot with the GPEM attached to their right thigh, as depicted in Figure 7.

During the test, both the gait phase estimated by the GPEM and the ground reaction force from the treadmill were recorded. Additionally, raw data, including 3-axis acceleration and 3-axis angular velocity, were recorded for validation purposes in the application of other gait phase estimation methods. The data from the GPEM were transmitted to the robot controller attached to the user’s back using the $I^{2}C$ communication protocol. Subsequently, the acquired data were sent to the computer system for recording purposes via the UART communication protocol. To synchronize the data from the GPEM and the treadmill, subjects were instructed to impact the ground using their right foot at the beginning of each trial. The ground reaction force (GRF) data was used to establish the ground truth for gait events, specifically for detecting heel strike and toe-off. Heel strike was considered to occur when the vertical GRF exceeded 10% of body weight (BW), while toe-off was identified when the vertical GRF fell below 10% of BW.

The standard deviations of the gait phase at certain gait events were selected as performance metrics. These metrics indicate robustness with respect to walking speeds. The proposed gait phase estimation method was evaluated using these metrics and compared to conventional methods, including the phase portrait method using a low-pass filter and the time-based estimation method.

### 4.2. Validation of Proposed Gait Phase Estimation Algorithm across Various Gait Speeds

To demonstrate the consistency of gait phase across different walking speeds, participants were instructed to walk for one minute at speeds ranging from 0.6 m/s to 1.6 m/s, with 0.2 m/s increments, covering the entire range of walking speeds.

**Figure 8:**
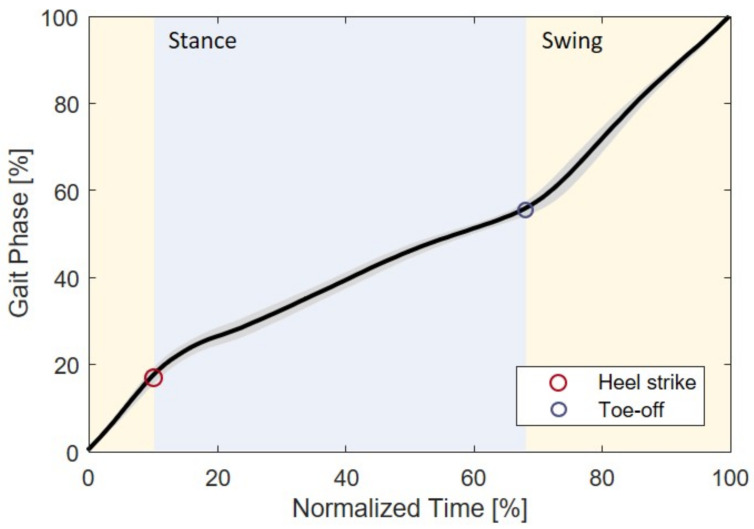
Gait phase estimated by the GPEM over normalized time with various walking speeds. Black solid line and shaded area indicate the mean and standard deviation of the gait phase, respectively.

Figure 8 presents the estimated gait phase of a participant over normalized time, which is the time elapsed in the current stride divided by the gait period. The data in the figure encompass the entire speed range for a subject. The solid line and shaded area indicate the mean and standard deviation, respectively. It is evident that the gait phase exhibits a continuous and monotonic pattern with minimal deviation. On average, heel strike and toe-off occurred at 17.24% and 55.14%, respectively. It is noteworthy that the distinction between stance (heel strike to toe-off) and swing (toe-off to heel strike) is clearly discernible from the slope of the graph.

**Figure 9:**
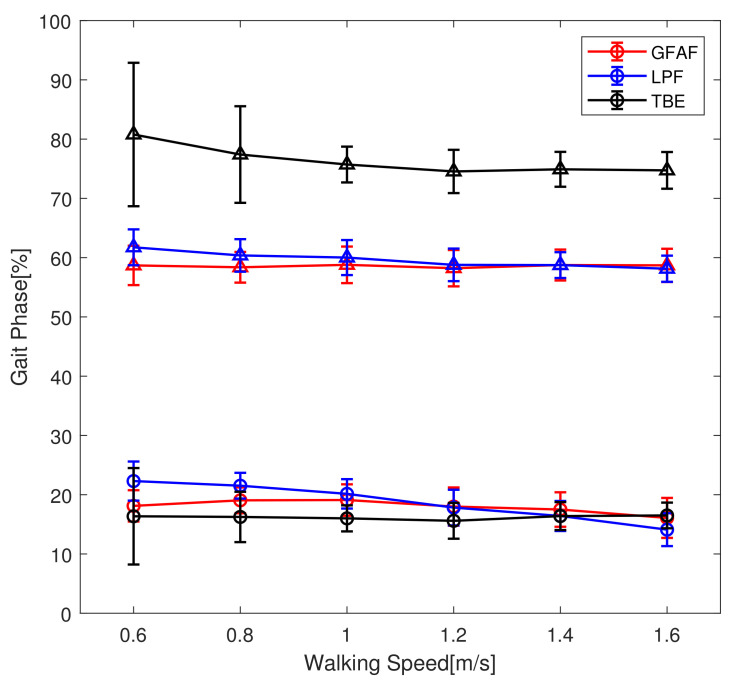
The gait event detection across the walking speed using three methods: the proposed method (red), the phase portrait with a low-pass filter (blue), and time-based estimation (black). Circles and triangles indicate heel strike and toe-off, respectively.

The gait phase should not only be monotonic and continuous but also detect certain gait events repetitively in every stride. Therefore, the standard deviations of the gait phase at the certain gait events, i.e., the heel strike and toe-off, are adopted as performance metrics for gait phase estimation. The performance of the proposed method using the GFAF was compared with time-based estimation (TBE) and the phase portrait using the low-pass filter (LPF). TBE used the raw kinematic data from the previous stride. Figure 9 shows the average and standard deviation of the gait phase at the gait events across different walking speeds, representing data from all subjects. When a low-pass filter was applied, the gait phase at the same gait event gradually decreased as walking speed increased. This is believed to be due to the increasing phase delay caused by the filter with higher walking speeds. The TBE method exhibited a larger deviation, especially at the low walking speeds where slow walking reduced gait stability and caused variations in gait period. In contrast, when GFAF was applied, a consistent gait phase corresponding to the same gait event was observed regardless of walking speed.

**Figure 10:**
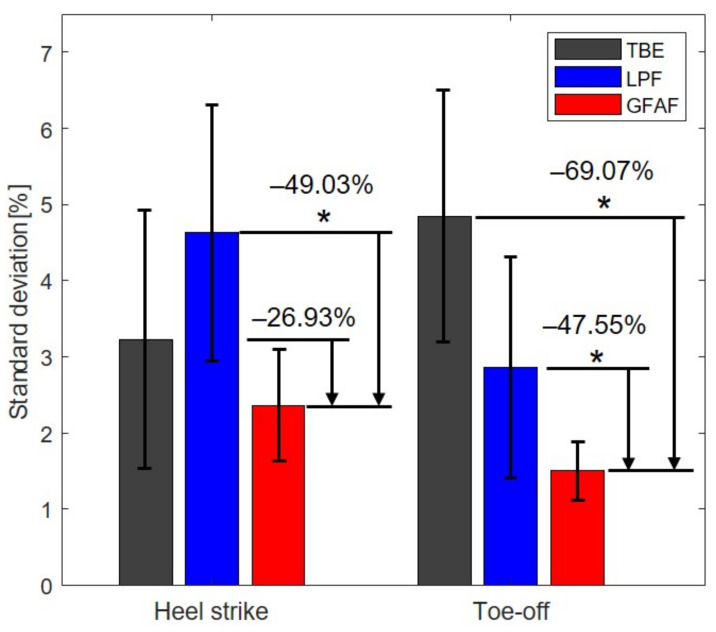
The standard deviation of the gait phase at certain gait events using three methods: the proposed method (red), the phase portrait with a low-pass filter (blue), and time-based estimation (black). The proposed method outperformed the conventional methods. The asterisks indicate statistical differences (p<0.05).

Figure 10 indicates the standard deviation of the gait phases at heel strike and toe-off using the three methods. It shows that the proposed method outperformed the other methods. The proposed method reduced the standard deviations of the gait phase at heel strike and toe-off by 49.03% (p<0.05) and 69.07% (p<0.05) compared to the low-pass filter, respectively. Additionally, compared to TBE, the proposed method reduced them by 26.93% (p=0.15) and 47.55% (p<0.05), respectively.

Table 1 presents the mean and standard deviation of the gait phase at heel strike and toe-off obtained using the three estimation methods. For each subject, over 400 strides were analyzed, encompassing all walking speeds. Heel strike was observed to occur between 11.36 and 21.51% of the gait phase, while toe-off took place between 55 and 63%. The variability in the gait phase, where heel strike and toe-off events occur, differs among subjects due to their individualized and optimized gait characteristics developed over their lifetime. While the proposed method did not significantly alter the mean values of the gait phase for each participant, it effectively reduced the standard deviation in all cases compared to when the low-pass filter was applied. This outcome indicates the superior precision of the proposed method in estimating the gait phase across the entire range of walking speeds.

### 4.3. Validation of Proposed Gait Phase Estimation Algorithm over Varying Gait Speeds

**Figure 11:**
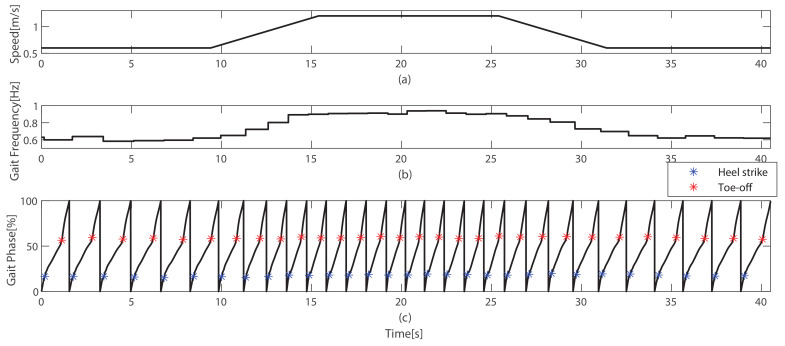
Real-time performance of gait phase estimation. (**a**) Treadmill speed profile, which contains constant speed and acceleration/deceleration. (**b**) Estimated gait frequency obtained using (7). (**c**) The result of the gait phase estimation across varying speeds.

To demonstrate the estimation of gait phase in real-time applications, walking test across varying walking speeds was conducted. A predefined speed profile was implemented on the treadmill, as illustrated in Figure 11a. The speed profile included constant speeds of 0.6 m/s and 1.2 m/s, along with an acceleration of 0.1 m/s${}^{2}$ and a deceleration of −0.1 m/s${}^{2}$. The test video is accessed via the following link: https://youtu.be/RNbOPfUzdvk (accessed on 27 August 2023).

Figure 11b shows the real-time estimation of gait frequency using (7), which exhibited a proportional relationship with the walking speeds. The gait phase estimation results using the GFAF, as well as the occurrence of gait events (heel strike and toe-off) for a single subject during walking, are depicted in Figure 11c. The heel strike was detected at 17.63±1.28% of the gait phase, while the toe-off occurred at 59.17±1.15%. The figure clearly demonstrates that the gait phase was continuously estimated, and the gait events consistently occurred at specific gait phases, not only during the constant speed section but also during the accelerated and decelerated speed sections.

## 5. Application to Gait Assistive Robots

The GPEM offers several advantages for controlling gait assistive wearable robots. Firstly, the GPEM can be applied to various form factors of robots. Since the GPEM only needs to be attached to the thigh without additional mechanical constraints, it can be used not only for exoskeleton robots but also for soft wearable robots. Moreover, due to its placement at the proximal part of the lower limb, the GPEM can be employed to assist various target joints such as the hip, knee, and ankle. This flexibility allows for adaptable and versatile control of wearable robots, accommodating different locomotion requirements and assisting multiple joints simultaneously. Lastly, by detecting the gait phase instead of relying solely on robot control signals, the GPEM helps alleviate computational load for the robot controller. For instance, the algorithm embedded in GPEM, encompassing tasks such as data acquisition from the IMU, thigh angle estimation, and gait phase estimation, consumes approximately 25,000 ticks. In the context of a robot controller equipped with a 275 MHz MCU and operating under a 1ms sampling period, this corresponds to a utilization of 9% of its computational resources. Robot controllers require many computing resources, not just for the gait phase estimation algorithm but also for high-level control, motor control, error handling, and service algorithms. Therefore, distributing the computational load of gait phase estimation to an external device, i.e., GPEM, can help the management of the computational resources of robot controllers.

**Figure 12:**
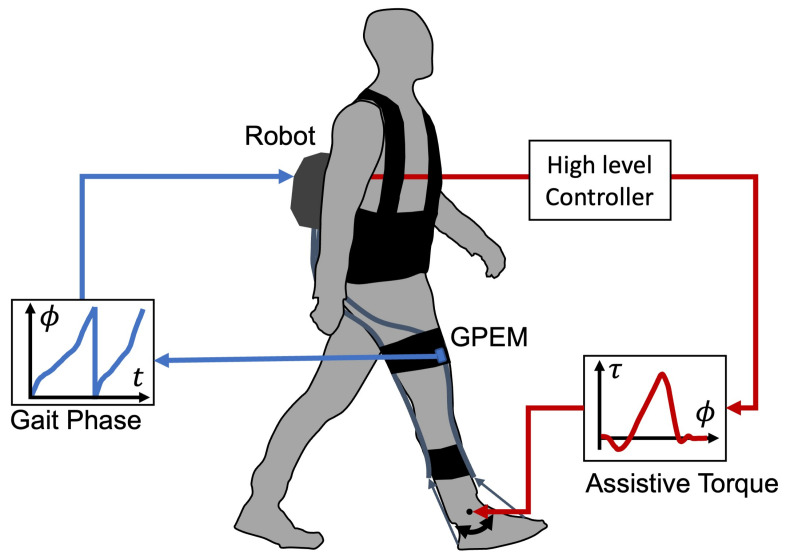
Application concept of the GPEM for the robot control.

Figure 12 illustrates the scenario of the gait phase-based control of the wearable robots. GPEM transmits the gait phase of the attached leg to the robot controller via CAN FD or $I^{2}C$ protocol. The GPEM converts the time domain to the gait phase domain. Based on the gait phase, the robot controller can generate the assistive torque trajectory on the gait phase domain. The assistive torque can be defined by various control strategies such as predefined torque generation [8,37], iterative control [10,38], human-in-the-loop optimization [3,9,39], and other advanced controllers.

## 6. Conclusions

This paper proposed a hardware of gait phase estimation module(GPEM) and its real-time algorithm. For real-world applications, the gait phase should be estimated in a continuous and monotonic manner over various walking speeds and accelerate/decelerate. A speed-adaptive online gait phase estimation algorithm enables precise gait phase estimation over constant speeds and dynamic speed changes. The proposed method was experimentally verified that the gait phase is estimated smoothly, continuously, and repetitively compared to the other methods such as the phase portrait method with a low-pass filter and the time-based estimation. The proposed algorithm is implemented in the GPEM to be applied versatilely to control the gait assistive robots without the additional computing burden.

In the following study, the GPEM will be utilized to control the various wearable robots. The robustness of gait phase should validate under the assistive torque applied. The assistance profile will be determined with regard to the gait phase from the algorithm and be applied in real time, as shown in Figure 12. Furthermore, to expand the range of applications for this algorithm, the authors are working on transforming the analysis domain from the sagittal plane to three-dimensional space. This expansion will allow the algorithm to be applied to a wider range of gait types, including circumduction gait. The communication protocol between GPEM and the robot controller will be evaluated. The packet loss and jitter will be used for the metrics of this evaluation [40,41].

## Figures and Tables

**Table 1:** Gait event detection results by three gait phase estimation methods for each subject.

Variables	Method	Gait Event	Subject 1	Subject 2	Subject 3	Subject 4	Subject 5	Subject 6	Subject 7	Subject 8	Subject 9	Subject 10	Subject 11
	GFAF	Heel strike (%)	21.55	19.72	16.29	18.73	14.48	16.65	14.05	18.61	13.29	17.24	11.36
		Toe-off (%)	62.47	55.60	59.62	59.21	56.23	59.00	56.89	53.94	59.31	55.14	63.01
Mean	LPF	Heel strike (%)	21.18	19.77	17.83	16.04	15.78	17.06	14.04	18.50	13.12	16.91	11.12
		Toe-off (%)	62.25	55.78	59.94	60.90	56.97	59.42	57.03	53.88	59.19	55.10	63.18
	TBE	Heel strike (%)	19.39	16.87	15.55	16.20	15.46	14.29	13.77	14.17	15.29	13.33	15.13
		Toe-off (%)	79.33	81.11	75.39	76.35	74.40	73.33	72.66	72.09	73.93	71.37	77.86
	GFAF	Heel strike (%)	1.52	1.64	1.88	1.52	3.08	2.19	2.59	3.04	2.41	3.81	2.33
		Toe-off (%)	1.31	1.61	1.34	1.67	1.09	2.24	1.44	1.27	0.96	1.58	1.99
Standard deviation	LPF	Heel strike (%)	3.03	2.29	4.19	8.74	4.32	3.98	4.66	5.03	4.24	6.11	4.31
		Toe-off (%)	1.72	1.88	3.41	4.35	1.56	3.18	3.36	1.75	1.34	6.13	2.83
	TBE	Heel strike (%)	3.31	11.97	2.12	3.0	1.77	2.09	3.15	2.43	2.31	3.91	4.45
		Toe-off (%)	3.84	13.50	3.90	3.96	3.08	3.54	5.77	4.06	3.38	6.31	7.93

## Data Availability

Not applicable.
